# Intracranial neuroendocrine carcinoma with coexisting pituitary adenoma: a case report and comprehensive literature review

**DOI:** 10.3389/fonc.2025.1572068

**Published:** 2025-09-03

**Authors:** Wei Kang, Miaomiao Feng, Xintong Liu, Anle Duan, Beiyan Tang, Hong Yan, Qiang Dong, Xianjun Zhao, Lei Duan, Yawen Pan

**Affiliations:** ^1^ The Department of Neurosurgery, The Second Hospital of Lanzhou University, Lanzhou, Gansu, China; ^2^ The Second Hospital & Clinical Medical School, Lanzhou University, Lanzhou, Gansu, China; ^3^ Third People’s Hospital of Gansu Province, Lanzhou, Gansu, China; ^4^ The Department of pathology, The Second Hospital of Lanzhou University, Lanzhou, Gansu, China; ^5^ Key Laboratory of Neurology of Gansu Province, Lanzhou, Gansu, China

**Keywords:** neuroendocrine carcinoma, intracranial tumor, pituitary adenoma, clinical manifestation, pathology, treatment

## Abstract

Neuroendocrine carcinomas (NECs) originating from intracranial sites are exceedingly rare. Here, we present the case of a 29-year-old female with intracranial NEC coexisting with a pituitary adenoma (PA). The patient’s symptoms included intermittent headaches, visual impairment, and endocrine abnormalities. Imaging studies revealed a large lobulated mass in the left frontal lobe and a mass lesion in the sellar region. Laboratory investigations indicated elevated levels of prolactin (PRL) and growth hormone (GH). Surgical resection of the frontal lobe tumor was performed initially, followed by neuroendoscopic transsphenoidal resection of the sellar lesion. The case underscores the diagnostic complexity of intracranial NEC due to its nonspecific clinical and radiological manifestations. Surgery remains the primary treatment modality, with adjunctive radiotherapy and chemotherapy. Further research is essential to enhance diagnostic accuracy and refine treatment strategies for intracranial NEC.

## 1 Introduction

Neuroendocrine carcinomas (NECs) are high-grade malignant neoplasms characterized by neuroendocrine differentiation and a high proliferative index, often classified as grade 3 according to the World Health Organization (WHO) criteria (1). While the gastrointestinal tract and lungs are the most common primary sites, NECs can theoretically arise in any organ (2). Primary intracranial NECs are exceptionally rare, and most NECs found within the central nervous system(CNS) represent metastases from extracranial sites (3).

Due to their rarity, the clinical and pathological features of intracranial NECs remain poorly defined. These tumors are often challenging to diagnose, especially when no primary lesion is identified on systemic imaging (4). Immunohistochemistry (IHC) plays a crucial role in determining the potential tissue of origin, particularly in cases of NEC of unknown primary (NEC-CUP) (5). Among organ-specific markers, SATB2 is increasingly recognized as a sensitive indicator of colorectal origin, even in poorly differentiated NECs (6).

Even more uncommon is the coexistence of an intracranial NEC with a pituitary adenoma (PA). To the best of our knowledge, this constellation has rarely been reported in the literature. The presence of two distinct intracranial neoplasms in the same patient introduces diagnostic uncertainty and complicates therapeutic planning, particularly when one of them is an aggressive and high-grade malignancy.

In this report, we present a unique case of a 29-year-old woman with a high-grade intracranial NEC accompanied by a sparsely granulated lactotroph PA. The NEC exhibited immunophenotypic features suggestive of colorectal origin, despite the absence of a detectable primary lesion on colonoscopy or Positron emission tomography–computed tomography(PET-CT). This case highlights the challenges in diagnosing NEC-CUP and emphasizes the importance of comprehensive pathological evaluation and multidisciplinary discussion in managing such rare and complex presentations.

## 2 Case presentation

A 29-year-old female was admitted with complaints of intermittent headache accompanied by visual impairment for 2 weeks; the headache was aggravated by nausea and vomiting for 2 days. Upon admission, physical examination revealed decreased vision in both eyes (Oculus Dexter: 0.5, Oculus Sinister: 0.6) and a visual field defect on the left temporal side—No abnormal enlargement of the hands, feet, or facial features. No protrusion of the jaw, tongue hypertrophy, or spontaneous nipple discharge was observed. No other significant abnormalities were detected. The patient has been generally healthy in the past but reported irregular menstruation for the past 3 years and amenorrhea for the past 4 months (**Table 1**).

**Table 1:** Timeline for patient care.

Date	Event	Details
Initial Presentation	Symptoms	Intermittent headaches, visual impairment, endocrine abnormalities
Diagnostic Imaging and Laboratory Findings	Imaging Results	Large lobulated mass in the left frontal lobe, occupying the lesion in the sellar region
	Laboratory Results	Elevated PRL and GH levels
Surgical Interventions	Initial Surgery	Date: 2021-11-12 Procedure: Surgical resection of the frontal lobe tumor Outcome: Successful removal of the frontal lobe tumor
	Second Surgery	Date: 2021-11-30 Procedure: Neuroendoscopic transsphenoidal resection of the sellar lesion Outcome: Successful removal of the sellar region tumor
Pathological Findings	Frontal Lobe Tumor	Diagnosis: NEC
	Sellar Region Tumor	Diagnosis: Sparsely granulated GH-PA
Postoperative Follow-Up	3 Months Post-Surgery	Findings: MRI showed no recurrence of intracranial and sellar region lesions Hormone Levels: Significantly decreased PRL and GH levels
	6 Months Post-Surgery	Findings: MRI revealed recurrence of the intracranial lesion at the original site, no recurrence in the sellar region Hormone Levels: Normal PRL and GH levels, regular menstruation
Further Treatment Recommendations	Postoperative Recommendations	Recommendation: Due to the high malignancy and recurrence rate of the intracranial lesion, radiotherapy and chemotherapy were recommended Patient Decision: Did not pursue further treatment for personal reasons, lost to follow-up 6 months later

## 3 Investigations

### 3.1 Imaging studies

**Figure 1:**
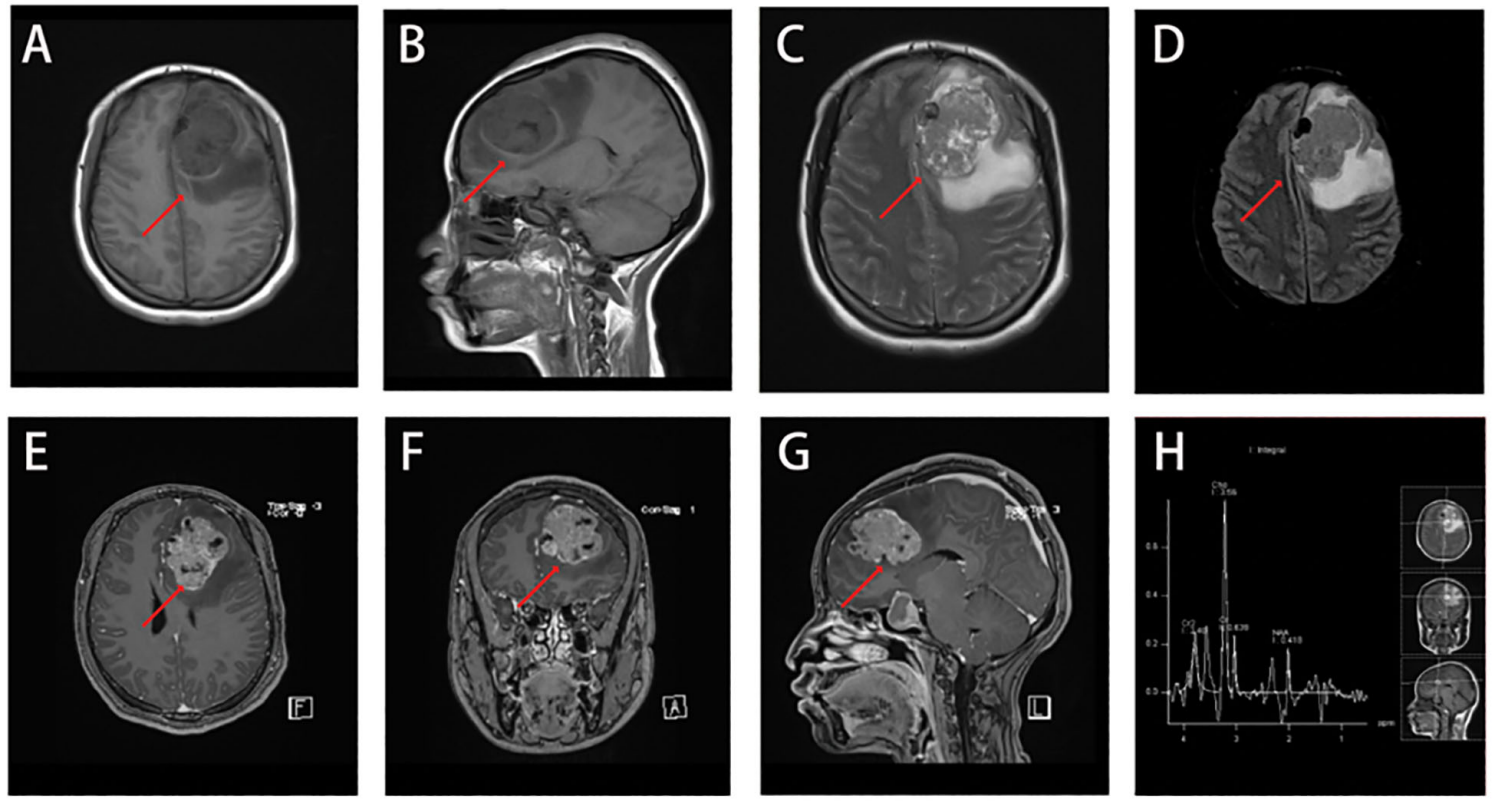
Irregularly shaped occupying lesion in the left frontal lobe, consider glioma or vascular ependymoma, metastatic tumor not excluded. **(A-C)** A large lobulated mass was seen in the left frontal lobe, measuring about 41×35×54 mm, with a slightly long T1 and long T2 signal, and the internal signal was not homogeneous. And there were multiple patches of long T1 and long T2 signal, nodular long T1 and short T2 signaling is seen in the medial margins. **(D)** Slightly high signal in FLAIR, the medial margin of FLAIR showed low signal, surrounded by edema. **(E-G)** The enhancement scan showed obvious heterogeneous enhancement, with more obvious enhancement of the medial margin of the nodule, and the midline structure was shifted to the right. **(H)** MRS suggested that the Cho peak in the area of interest was obviously elevated, and the NAA peak was reduced and partially disappeared.

Magnetic resonance imaging (MRI) (**Figure 1**) of the brain revealed a large, irregularly shaped lobulated mass located in the left frontal lobe, measuring approximately 41 × 35 × 54 mm. The tumor was situated within the subcortical white matter and extended close to the gray-white matter junction. It did not involve the skull bone or the dura mater directly, but the mass effect led to compression of adjacent cortical structures and midline shift to the right.

On imaging, the lesion demonstrated slightly prolonged T1 and T2 signal intensity with heterogeneous internal signals and multiple patchy areas. FLAIR sequences revealed mild hyperintensity with surrounding perilesional edema. DWI showed iso-intensity. The inner margin exhibited nodular areas with prolonged T1 and shortened T2 signals, suggestive of necrotic or hemorrhagic components. Gadolinium-enhanced images demonstrated marked heterogeneous enhancement, with prominent enhancement at the inner margin. Magnetic resonance spectroscopy (MRS) showed a significantly elevated choline (Cho) peak and reduced or absent N-acetyl aspartate (NAA) peak, findings consistent with high-grade malignant tumor pathology.

**Figure 2:**
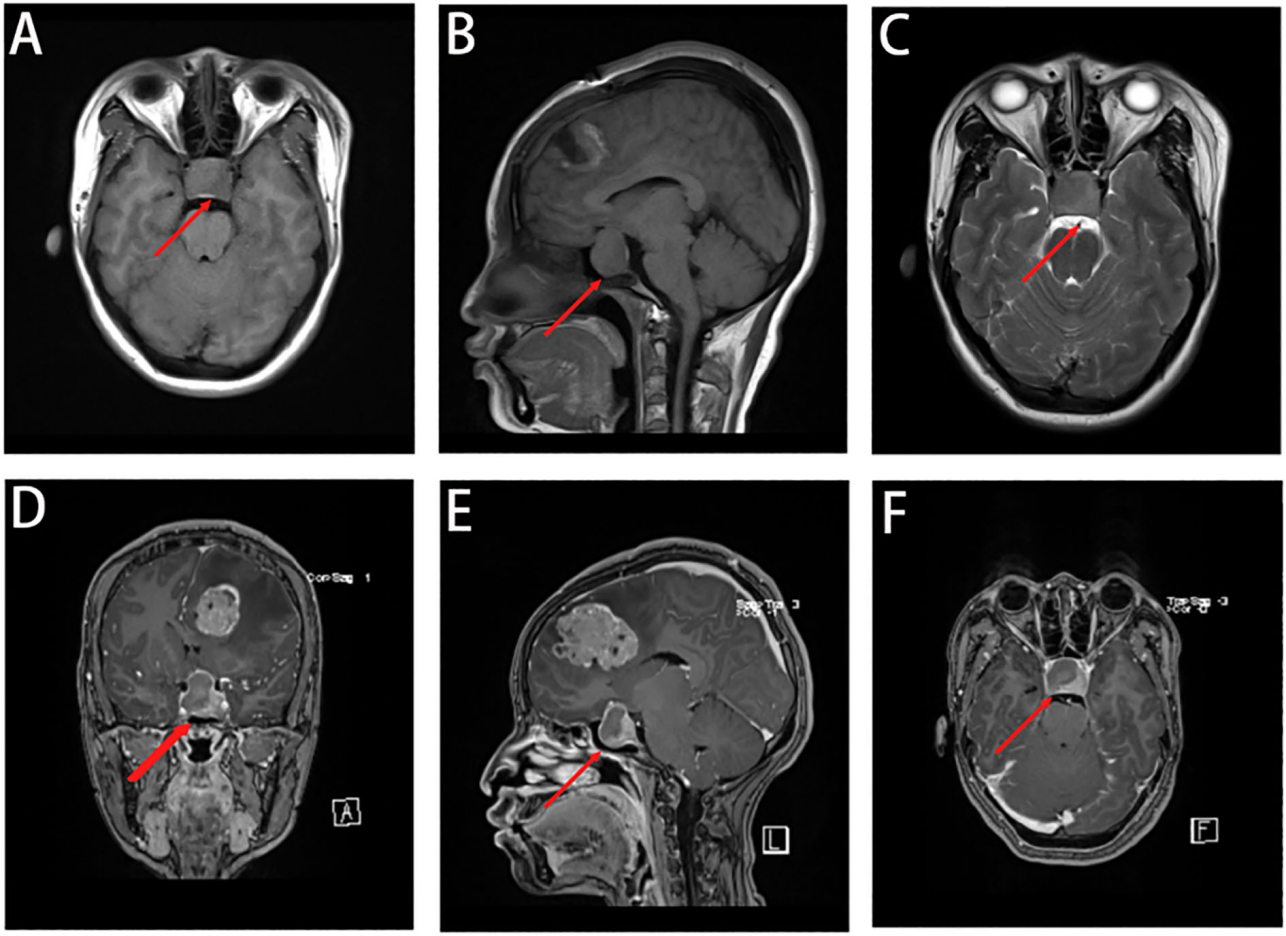
Occupying lesion in the saddle region, consider pituitary macroadenoma, encircling bilateral cavernous sinuses, with elevation of the optic cross. **(A-C)** A space-occupying lesion was seen in the saddle region, showing an isotropic T1 and isotropic T2 signal shadow, measuring about 25 × 32 × 20 mm. **(D-F)** The enhancement scan shows mild flocculent enhancement in the center, marked enhancement in the rim ring, and bilateral cavernous sinus encircling, and the optic cross is compressed and elevated.

A well-defined mass was centered in the sellar region, measuring approximately 25 × 32 × 20 mm (**Figure 2**). The lesion expanded both superiorly and laterally, leading to elevation and compression of the optic chiasm. The mass extended into both cavernous sinuses, encasing the internal carotid arteries bilaterally. There was no evidence of direct invasion into the skull base or adjacent bone structures.

The lesion demonstrated iso-intensity on T1- and T2-weighted images, with mildly increased signal intensity on FLAIR sequences. Post-contrast enhancement demonstrated mild central flocculent enhancement and prominent ring-like peripheral enhancement. The tumor’s imaging characteristics and anatomical localization were consistent with a pituitary macroadenoma with suprasellar and parasellar extension.

### 3.2 Laboratory investigations

**Table 2** lists the abnormal values of multiple pituitary hormone tests for this patient before and after surgery. PRL significantly decreased after surgery and returned to normal levels by 3 months post-op. Menstruation normalized 6 months post-op. Luteinizing Hormone(LH) was low before surgery but returned to normal 1 month post-op. GH was significantly elevated before surgery and gradually decreased, returning to normal 6 months post-op. Insulin-like Growth Factor 1(IGF1)showed a gradual downward trend after surgery.

**Table 2:** Hormonal levels and growth factors in a patient pre- and post-surgery.

Item	Normal Range	Admission	Post-op	1 Month Post-op	3 Months Post-op	6 Months Post-op
PRL	Non-pregnant women (2.80-29.2) (ng/mL)	138.60	84.66	122.85	8.18	5.76
LH	Follicular phase (1.90-12.50) (mIU/mL) Ovulatory phase (8.70-76.30) Menopausal phase (23.00-116.30)	0.957	0.040	4.360	2.980	1.920
GH	(0.06-5.00) (ng/mL)	36.15	7.48	7.46	5.79	3.80
IGF1	(71.00-234.00) (ng/mL)	–	687	516	513	456

## 4 Diagnosis and treatment process

Upon admission, the patient underwent comprehensive routine examinations, with no apparent contraindications for surgery identified. Due to significant edema and mass effect in the left frontal lobe, accompanied by obvious symptoms of increased intracranial pressure and a risk of brain herniation formation, priority was given to the resection of the frontal lobe tumor. Intraoperatively, the tumor was identified within the parenchyma of the left frontal lobe, presenting as irregular in shape, soft in texture, gray-red in color, highly vascularized, with arachnoid interspaces between the surrounding brain tissues, and without obvious basal attachment. Calcifications were observed within the tumor. The tumor was grossly resected en bloc under the microscope. The patient recovered well postoperatively.

**Figure 3:**
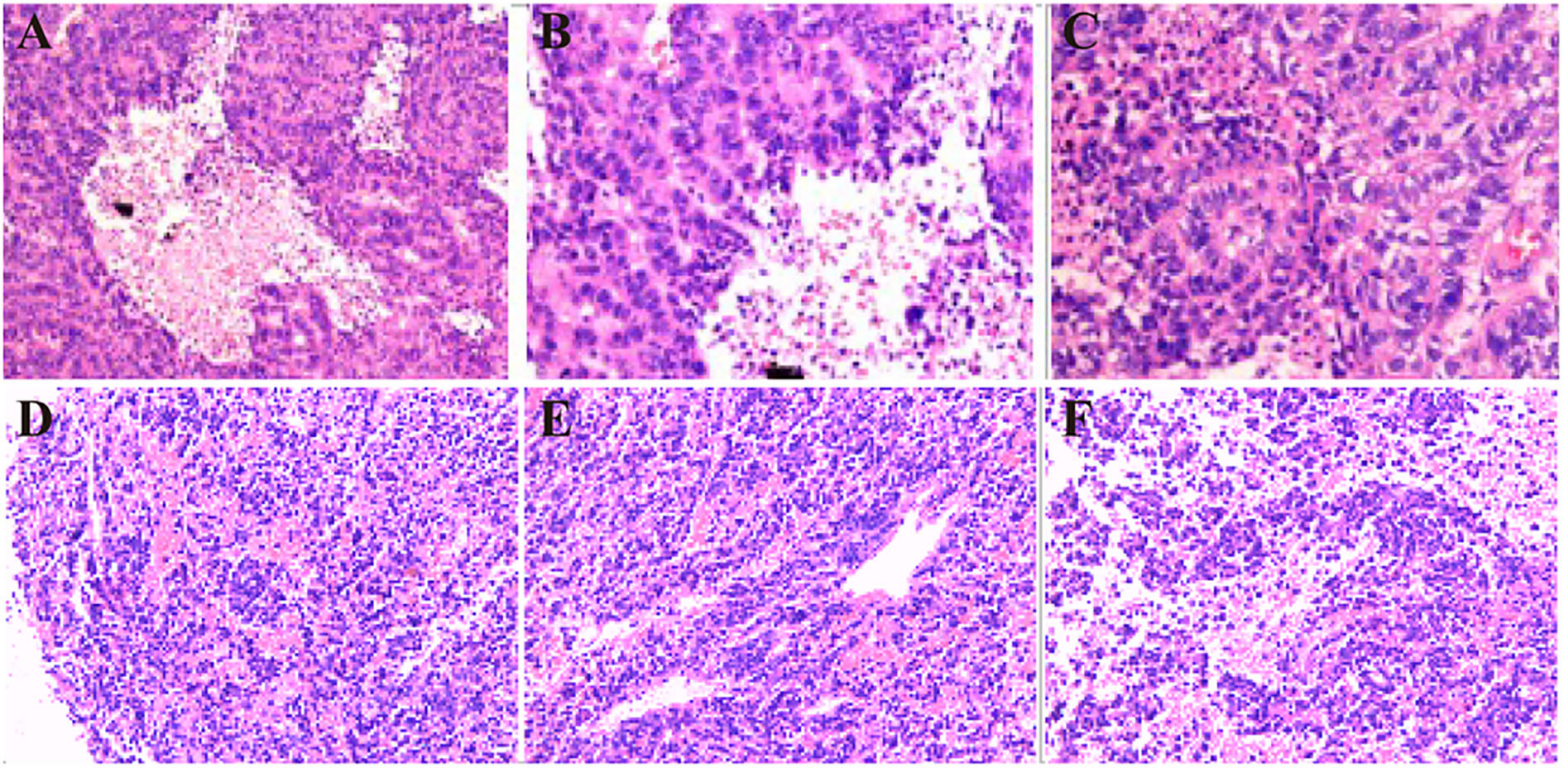
Pathologic findings of NEC and PA. **(A-C)** Sparse granular GH-PA. **(D-F)** Tumor cells are arranged in a ribbon-like pattern; some areas form pseudo-daisy mass-like structure around blood vessels, cytoplasm is eosinophilic, nucleoplasma ratio is enlarged, nucleus is short spindle or irregular, a few cells have obvious nuclear anomalies, nuclear schizophrenia is >20/10HPF, and multiple necrotic foci are seen (Intracranial NEC).

Histopathological analysis of the left frontal lobe lesion revealed a poorly differentiated NEC with high-grade features (**Figure 3**). Hematoxylin and eosin (H&E) staining demonstrated tumor cells arranged in ribbon-like and pseudorosette patterns, with eosinophilic cytoplasm, prominent nuclear pleomorphism, and a mitotic rate exceeding 20/10 high-power fields. Extensive necrosis was observed.

IHC demonstrated that the tumor cells were diffusely positive for CD56 and focally positive for Synaptophysin, supporting neuroendocrine differentiation (7). Chromogranin A was negative. Notably, strong nuclear expression of SATB2 was detected, a sensitive marker suggestive of colorectal origin (8). The tumor cells were also positive for CKpan (focal), CK8/18 (weak), and EMA (partial). The Ki-67 proliferation index was approximately 60%, indicating a grade 3 neuroendocrine neoplasm (9). Additional immunostains were negative for TTF-1, CDX2, CK7, CK20, PAX8, GATA3, GFAP, Olig2, NeuN, and LCA, helping exclude pulmonary, thyroid, breast, renal, and primary CNS origins (9–13).

**Figure 4:**
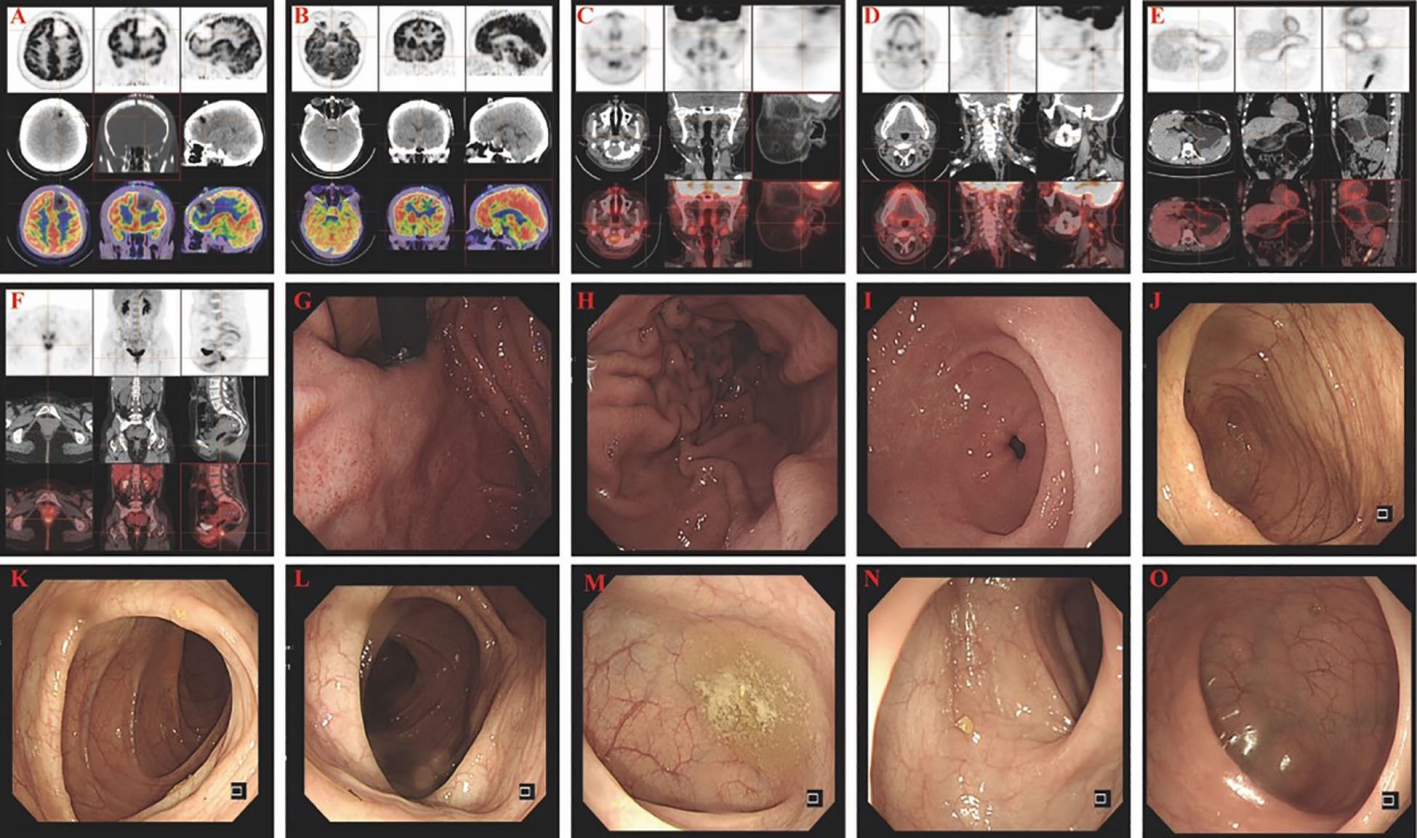
PET-CT, upper gastrointestinal endoscopy, and colonoscopy findings. **(A)** PET-CT image showing increased FDG uptake at the left frontal scalp surgical site (SUVmax: 12.6), consistent with postoperative inflammation. The left frontal lobe resection cavity demonstrates postoperative changes with hypometabolic areas suggestive of hemorrhage, fluid collection, and perifocal edema, resulting in compression of the left frontal horn and adjacent sulcal effacement. **(B)** A hypermetabolic lesion in the sellar region (27 × 19 × 25 mm) protruding into the suprasellar cistern and third ventricle, with increased FDG uptake (SUVmax: 12.4), causing mild third ventricular compression. **(C)** A left parotid nodule (short axis ~8 mm) with mildly increased FDG uptake (SUVmax: 3.3). **(D)** Multiple enlarged cervical lymph nodes are seen bilaterally in level Ib to III, with some showing increased FDG activity (SUVmax: 4.6); the largest is located in the left level II (short axis ~10 mm). **(E)** PET-CT image of the stomach shows uneven, diffuse FDG uptake (SUVmax: 3.9) without obvious wall thickening, consistent with chronic gastritis. **(F)** Increased FDG uptake is observed in the anorectal region and sigmoid colon (SUVmax: 3.4–5.3), although the perirectal fat planes remain preserved. **(G–I)** Upper gastrointestinal endoscopy reveals chronic atrophic gastritis involving the gastric fundus **(G)**, body **(H)**, and antrum **(I)**, with no evidence of ulceration or malignancy. **(J–O)** Colonoscopic examination of the ileocecal region **(J)**, ascending colon **(K)**, transverse colon **(L)**, descending colon **(M)**, sigmoid colon **(N)**, and rectum **(O)** shows no mucosal lesions or neoplastic abnormalities.

Further review by the institutional pathology department, with supplementary staining including Vimentin (+), INI-1 (retained), NKX2.2 (+), Desmin (–), and CD99 (–), supported the diagnosis of a metastatic high-grade NEC. The presence of SATB2 positivity, combined with the IHC profile and histological features, strongly suggested a colorectal origin, although colonoscopy and PET-CT (**Figure 4**) failed to identify a definitive primary tumor. Given the uncertain origin and aggressive features, a neuro-oncology pathology consultation was recommended. The final interpretation favored a diagnosis of metastatic colorectal G3 NEC.

Further investigations revealed chronic atrophic gastritis with erosion on esophagogastroduodenoscopy (**Figure 4**), while colonoscopy showed no significant abnormalities. Whole-body PET-CT suggested a sellar lesion, with increased metabolic activity in one lymph node in the left cervical region, and no other significant tumor-related changes were observed. Due to significant visual impairment, amenorrhea, and elevated levels of PRL and GH, which were suspected to be related to the sellar lesion, there was a clear indication for surgery. The nature of the sellar region tumor helps to clarify whether the frontal lobe tumor is related to the pituitary tumor. The sellar region tumor has clear surgical indications, and a transsphenoidal pituitary tumor resection was performed under general anesthesia using neuro-navigation and neuro-endoscopy. The surgery was successful, and the patient reported significant improvement in vision postoperatively. Postoperative pathology of the sellar region tumor indicated a sparsely granulated Growth hormone-secreting PA (GH-PA) (**Figure 3**).

**Figure 5:**
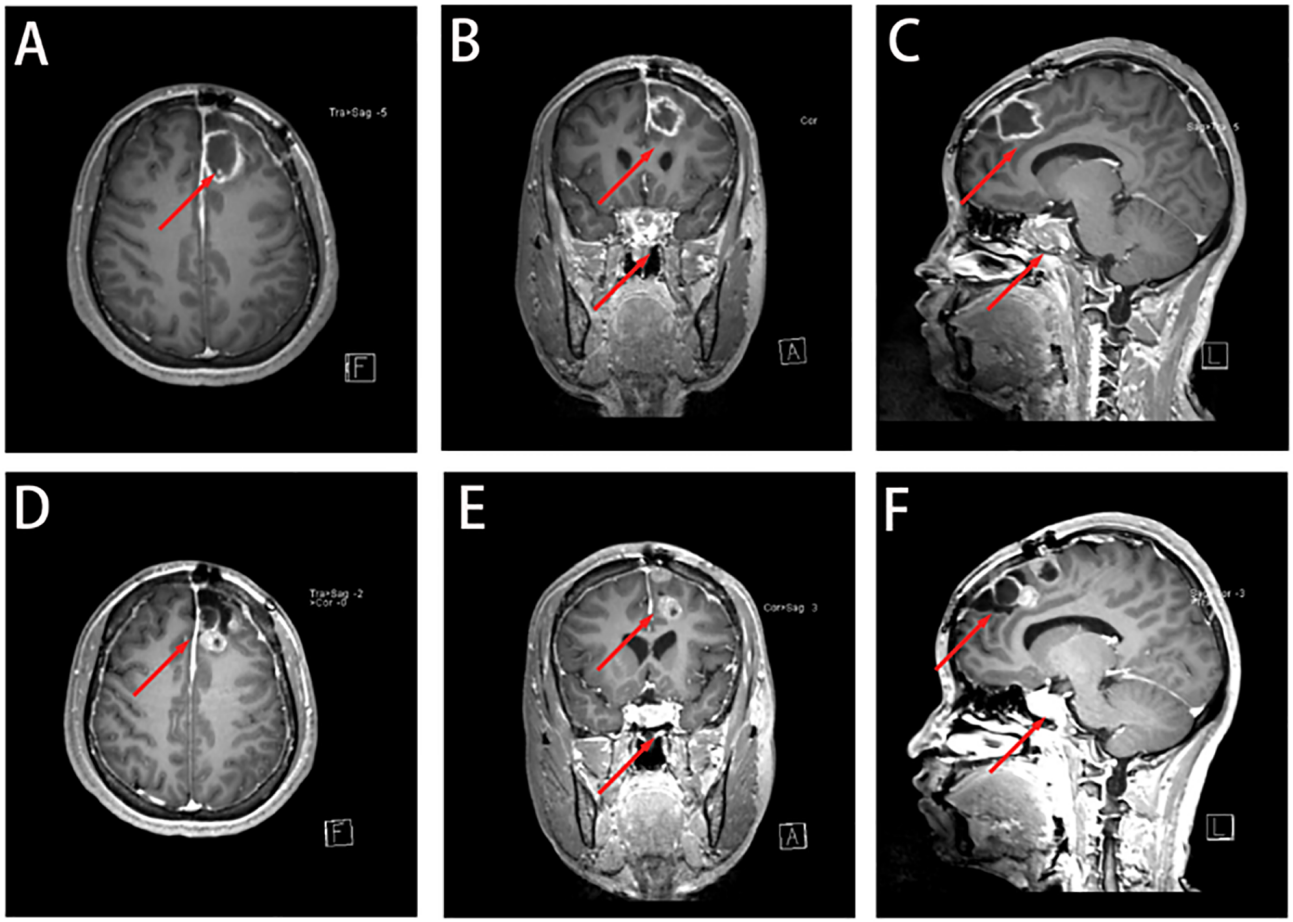
Follow-up information. **(A-C)** The tumor did not recur at the 3-month postoperative follow-up. Visible enlargement of the sella turcica, depression underneath the sella, ring-enhancing lesion, and elevation of the optic chiasm; structural disorder of the left frontal lobe, ring-enhancing lesion visible on the left side of the falx cerebri, adjacent thickening and enhancement of the meninges. Midline structures are centrally located. No recurrence of intracranial and sellar tumors. **(D–F)** The tumor did not recur at the 6-month postoperative follow-up.

A follow-up MRI 3 months post-op demonstrated no recurrence of the intracranial and sellar region lesions (**Figure 4**), and pituitary hormones PRL and GH had significantly decreased compared to pre-surgery levels. However, 6 months post-op, an MRI revealed a recurrence of the intracranial lesion at the original site (**Figure 5**), but no recurrence in the sellar region, with PRL and GH levels normal and menstruation regular (**Table 2**). Given the high malignancy and recurrence rate of the intracranial lesion, postoperative radiotherapy and chemotherapy were recommended. However, the patient did not pursue further treatment for personal reasons and was lost to follow-up 6 months later.

## 5 Discussion and literature review

NECs are rare, highly aggressive malignancies, particularly when arising in the CNS (14). While the majority of reported intracranial NECs represent metastases from extracranial origins—most commonly the lungs or gastrointestinal tract—primary intracranial NECs are exceedingly rare, and their clinical profiles remain insufficiently characterized (15). The case presented herein is made even more unusual by the concurrent diagnosis of a PA, which, to our knowledge, has rarely been reported in the literature (16).

### 5.1 Coexisting tumors in the sellar and extrasellar regions: literature context and comparison

A limited number of studies have reported cases involving metastatic carcinomas to the sellar region. For example, a clinical analysis of six cases documented metastases in the pituitary region originating from various primary sites, emphasizing the need for careful differential diagnosis when evaluating sellar lesions (17). In our case, while the sellar mass was ultimately diagnosed as a sparsely granulated GH-PA, the coexisting intracranial NEC raised initial concerns regarding potential metastatic involvement or a “collision” phenomenon.

Other reports, such as the case of xanthomatous hypophysitis (18) and ectopic thyrotropin-secreting PA (19), further illustrate the wide spectrum of unusual sellar or parasellar lesions that may coexist with or mimic neoplastic processes. These findings highlight the importance of comprehensive histopathological assessment, even when imaging is suggestive of benign lesions like PAs.

### 5.2 Biological considerations for tumor coexistence: Coincidence or pathogenic link?

The simultaneous presence of a high-grade intracranial NEC and a PA in a young patient is exceptionally rare, raising the question of whether this represents a mere coincidence or a biologically linked phenomenon.

One possibility is incidental coexistence, as multiple primary brain tumors—though uncommon—have been previously reported (20, 21). Alternatively, hormonal influence from the PA (e.g., excess GH and PRL) may have created a microenvironment favorable to tumorigenesis, although direct evidence in NEC is lacking (22–24). A shared genetic predisposition or origin from common progenitor cells is another hypothesis, especially in younger patients, and may warrant molecular investigation (25).

In the absence of systemic malignancy and with negative PET-CT and colonoscopy, we consider this a case of dual primary tumors rather than metastasis from the pituitary lesion.

### 5.3 Diagnostic challenges of NEC-CUP

The diagnostic process for NEC-CUP remains complex (26). In our case, IHC played a pivotal role in narrowing down the most likely origin of the tumor. The tumor exhibited diffuse CD56 and partial synaptophysin positivity, as well as a high Ki-67 index (>60%), which is consistent with grade 3 NEC (27–29). Importantly, strong SATB2 nuclear expression suggested a colorectal origin, even though CDX2 was negative and no colorectal lesion was identified on colonoscopy or imaging (6, 30).

The discordance between immunohistochemical findings and clinical imaging illustrates the central dilemma of NEC-CUP—diagnoses often hinge on surrogate molecular markers without definitive confirmation from primary tumor biopsy. SATB2 has emerged as a relatively specific marker for lower gastrointestinal origin, especially in poorly differentiated tumors, and may guide treatment decisions in the absence of histologically confirmed primaries (31).

Although next-generation sequencing (NGS) and DNA methylation profiling were not available in this case, their inclusion in future workups could offer greater diagnostic precision, possibly reclassifying these entities based on emerging tumor taxonomies.

### 5.4 Treatment implications, follow-up strategies, and future directions

Current treatment guidelines for NECs typically involve multimodal therapy, including surgery, radiotherapy, and platinum-based chemotherapy (32). In the present case, while surgical resection was successfully performed for both lesions, the patient declined adjuvant therapy and was lost to follow-up. This limits our ability to assess long-term outcomes and reinforces the need for early patient education and comprehensive oncologic planning.

Emerging strategies—including immunotherapy (e.g., PD-1/PD-L1 inhibitors) and targeted molecular therapies based on tumor-specific mutations—have shown promise in small-cell NECs and may be considered in similar intracranial presentations in the future (33). Additionally, frequent surveillance using advanced imaging (PET-MRI or multiparametric MRI) may help detect early recurrence in high-grade tumors, especially in cases where adjuvant therapy is refused or delayed (34, 35).

In conclusion, this case underscores the diagnostic and therapeutic complexity of intracranial NECs, particularly when coexisting with a second tumor type. It also illustrates the need for a systematic, multidisciplinary approach integrating histopathology, imaging, molecular diagnostics, and personalized treatment planning. Further accumulation of similar case reports and multi-institutional databases may help refine the classification and management of NEC-CUP and rare tumor co-occurrences in the CNS.

## 6 Conclusion

We report a rare case of intracranial high-grade NEC coexisting with a sparsely granulated GH-PA in a young female patient. The case highlights several diagnostic and therapeutic challenges, particularly in the context of NEC-CUP. Despite comprehensive imaging and endoscopic evaluation, no definitive extracranial primary lesion was identified, and the final diagnosis relied heavily on histopathology and immunohistochemical profiling, with strong SATB2 positivity suggesting a colorectal origin.

The concurrent presence of two distinct intracranial tumors underscores the importance of considering dual pathology in complex cases and illustrates the value of a multidisciplinary diagnostic approach. While surgical resection provided initial symptomatic relief and hormonal normalization, the patient’s refusal of adjuvant therapy limited long-term management.

This case contributes to the limited literature on intracranial NECs and their potential coexistence with pituitary tumors. It emphasizes the need for increased awareness, detailed pathological evaluation, and ongoing research into the molecular characteristics and optimal management strategies for such rare and aggressive neoplasms.

## Data Availability

The datasets presented in this article are not readily available because of ethical and privacy restrictions. Requests to access the datasets should be directed to the corresponding author.
